# How and to what extent did the Coventry City of Culture ‘City Host’ volunteer programme affect the volunteers’ mental wellbeing? A qualitative study

**DOI:** 10.1186/s12889-023-16862-7

**Published:** 2023-10-19

**Authors:** Maxine Whelan, Iman Ghosh, Lauren Bell, Oyinlola Oyebode

**Affiliations:** 1 Institute for Health and Wellbeing , Coventry University, Coventry, UK; 2 Warwick Evidence, University of Warwick, Coventry, UK; 3 Queen Mary University of London, Wolfson Institute of Population Health, London, UK

**Keywords:** Volunteering, Mental-wellbeing, Wellbeing, Social capital, Mixed-methods, Subjective wellbeing

## Abstract

**Background:**

A team of volunteers, known as City Hosts, were recruited to support UK City of Culture 2021 awarded to Coventry. City Hosts held various roles facilitating cultural event delivery and promoting a positive experience for visitors. This study aimed to (i) understand how and to what extent the volunteering programme impacted volunteer subjective wellbeing, and (ii) explore the mechanisms of change and intermediate outcomes between volunteering and subjective wellbeing.

**Methods:**

This qualitative study comprised inductive and deductive analysis of data collected through semi-structured interviews, conducted between December 2021–May 2022 with City Hosts. This was complimented with secondary qualitative analysis of free text responses within Monitoring and Evaluation data collected from City Hosts in surveys conducted in August and November 2021, and April 2022.

**Results:**

Approximately 180 City Hosts responded to the free text questions in each survey and 27 completed interviews. Analysis of data collected from City Hosts suggested positive wellbeing impacts from volunteering and supported theorised pathways to improved wellbeing. Strengths of the City Host programme included (i) facilitating the full range of mechanisms of change that mediate improved volunteer wellbeing, particularly promoting social connections and developing a strong role and group identity and (ii) flexibility around what volunteers do, how much, and how often.

**Conclusions:**

This study offers lessons for others designing volunteering programmes who wish to promote wellbeing among associated volunteers. We also offer evidence that exposure to culture may be one mechanism by which volunteering can improve wellbeing.

**Supplementary Information:**

The online version contains supplementary material available at 10.1186/s12889-023-16862-7.

## Background

**Fig. 1:**
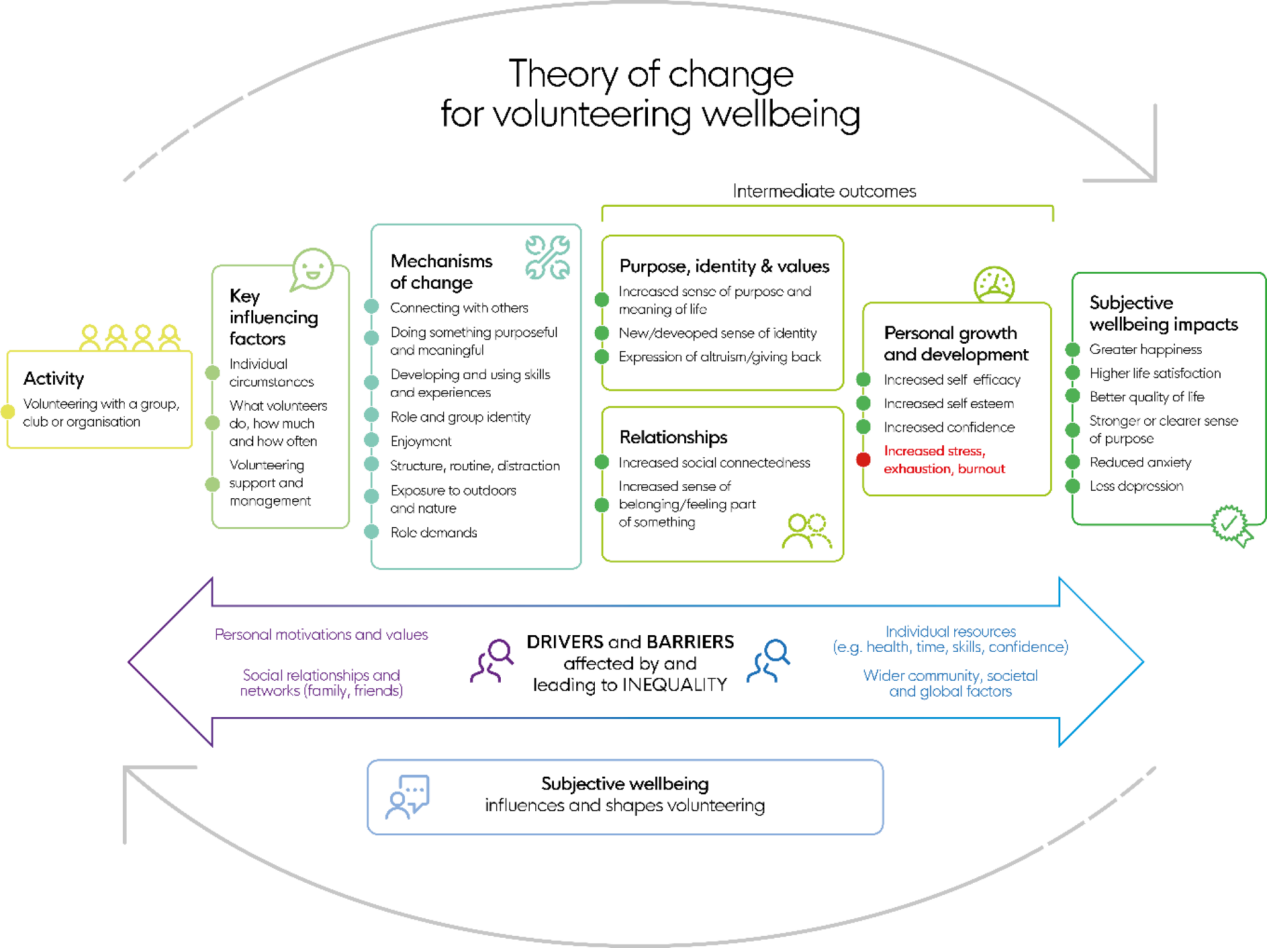
What Works Wellbeing Theory of Change for volunteer wellbeing. From Stuart, J., Kamerāde, D., Connolly, S., Ellis Paine, A., Nichols, G. and Grotz, J. (2020) *The Impacts of Volunteering on the Subjective Wellbeing of Volunteers: A Rapid Evidence Assessment*, What Works Centre for Wellbeing and Spirit of 2012 [online] What Works Centre for Wellbeing. Available at: https://whatworkswellbeing.org/wp-content/uploads/1920/10/volunteer-wellbeing-Oct-20_briefing.pdf

Subjective wellbeing is both a driver of, and consequence of, population health, as well as social and economic progress including prosocial behaviour [1]. A rapid review that included 158 studies in the synthesis, published between 2008 and 2020, reported that most evidence suggests formal volunteering (unpaid activity, undertaken through free-will, of benefit to others or the environment that is conducted through organisations, groups or clubs) is positively associated with subjective wellbeing. The review concluded that this is likely to be partly because volunteering can be good for wellbeing, but also possibly because those with higher levels of wellbeing are more likely to get involved in volunteering [2]. The review used the available evidence to identify several likely mediators of the volunteering-wellbeing relationship and synthesised these in a Theory of Change [Fig. 1]. The review also identified that some volunteering activities can lead to anxiety, stress or burnout, and two recent randomised controlled trials found no significant wellbeing benefits for older adults randomised to an intervention group in which volunteering was promoted [3, 4]. However, in one of the trials, those in the volunteer group who increased their volunteering did show benefits [3]. The potential for volunteering programmes to help, hinder or have no effect on the subjective wellbeing of those taking part needs further investigation.

The ‘UK city of culture’ is a title given to a city in the United Kingdom every four years, by the Department for Digital, Culture, Media and Sport (DCMS). Cities and local areas enter a competitive process to attain the designation. For a period of one calendar year, the successful city (or local area), awarded the ‘UK City of Culture’ hosts a number of cultural activities. In December 2017, Coventry was awarded the title of UK City of Culture. For UK City of Culture 2021, the year was delayed and ran from May 2021-May 2022 (due to the COVID-19 pandemic), and an extensive partnership between public, private and civic organisation, two local universities, and citizens delivered the programme of events.

To support delivery of UK City of Culture 2021, Coventry City of Culture Trust in partnership with Coventry University Student’s Union and EnV, a Community Interest Company, recruited a team of volunteers. These volunteers were known as ‘City Hosts’. By the time the Coventry City of Culture 2021 team stopped recruiting new volunteers (30 June 2022), 4185 people had signed up to volunteer and 1515 were fully trained and deployed as City Hosts. Regardless of experience, all City Hosts were provided with a range of free training, including mandatory training and additional non-compulsory courses. A highly recognisable Coventry 2021 uniform was provided, which City Hosts were asked to wear while on the shift and an online platform was used for City Hosts to book shifts based on their interests and availability.

A huge range of shifts were available for City Hosts. Shifts included being stationed at key local transport hubs where they would welcome and orient visitors. Other shifts involved roaming Coventry and being a focal contact point for the public or being based at venues where UK City of Culture 2021 activities were scheduled, where City Hosts could provide information on artwork, installations and events. Additional training was also undertaken by some City Hosts who could then sign up to be a ‘Team Leader’, organising and supporting the City Hosts on that particular shift.

Volunteering as a City Host during the UK City of Culture is a specific kind of volunteering, which is sometimes known as episodic volunteering [5], or even more specifically, is a type of volunteering associated with a ‘mega-event’. A mega-event is usually used in a sporting context to include the Olympic and Commonwealth games, and is described as a large event that generates significant tourism, media interest and economic impact. Volunteering at mega-event can be different from other types of volunteering in that it is relatively intensive and involves large numbers of people [6]. The motivation to volunteer at a mega-event can also differ from other types of volunteering [7, 8]. Literature on this specific type of volunteering, as with the literature on volunteering in general summarised above, also finds that these volunteers report subjective wellbeing benefits in the short-term [6, 8, 9].

Commissioned by the UK City of Culture Trust, this study formed part of a larger piece of research which aimed to contribute towards the evidence for specific outcomes detailed within the UK City of Culture 2021 Story of Change [10]. Specifically, the UK City of Culture Trust sought to understand the influence of the City Host volunteering programme on the volunteers taking part. This paper reports how and to what extent the City Host volunteering programme impacted the subjective wellbeing of those volunteering, and explores the mechanisms of change and intermediate outcomes between volunteering in this programme and subjective wellbeing. In this study we particularly examine the impacts on volunteers that are presented as subjective wellbeing outcomes in Fig. 1i.e.: greater happiness, higher life satisfaction, better quality of life, strong or clearer sense of purpose, reduced anxiety and less depression. These impacts could also be described as mental wellbeing impacts, where mental wellbeing includes feeling good and functioning well and is one end of a continuum of mental health with the opposite end being mental illness (e.g.: anxiety and depression) [11]. Our aim was to draw generalisable findings about how volunteer programmes could be designed to maximise potential benefits and minimise potential harms for those volunteering.

## Methods

We used a convergent, parallel, mixed-methods approach to answer our research question, informed by a recent review of relevant evidence and resulting theory of change [Fig. 1] [2]. The theory of change was used to inform the design of this study and to aid qualitative data analysis. This theory of change was selected because it was informed by a recent review of relevant evidence at the time of this study. It is intuitive and was easily understood by the many stakeholders involved in commissioning the study or who may use the study findings.

While our original protocol and the final report to the funder included secondary quantitative analysis of survey data, there were several limitations to the survey data collected. This was mainly due to the impact of the COVID pandemic. We expected to conduct secondary analysis using Coventry Household Survey data as a control group which we could compare against data collected from City Hosts by the monitoring and evaluation team- having worked with the latter team to ensure some key comparable questions were included. However, the Coventry Household Survey data were collected during a period of severe social restrictions in January to March 2021, which studies both nationally and locally suggest had a clear negative impact on wellbeing [12, 13]. In contrast, the City Host surveys were conducted in August 2021-April 2022 when restrictions were lifted. In addition, there was a poor response rate to the monitoring and evaluation survey. This means we cannot be confident that any quantitative differences in wellbeing between City Hosts and the Coventry Household Survey are due to volunteering activities, or that they represent the experience of the general population of City Hosts. For this reason, we are presenting the qualitative data, analysis and findings only in this manuscript.

The study was reviewed and gained ethical approval from the University of Warwick Biomedical Sciences Research Ethics Committee (full ethical approval: BSREC 07/21–22).

### Research team reflexivity

The research team included four women, two holding PhDs (MW and OO) and two holding MScs (IG and LB). All four were working in local Universities at the time of the study. All members of the team had training and previous experience of conducting qualitative data collection.

The research team also visited City Host coffee mornings to meet City Hosts and discuss the on-going research. The team described the reasons for doing the research- to understand the impact of the volunteer programme on those taking part and to learn how these can be designed to maximise benefits for volunteers in the future. The team additionally solicited interest in participation in the semi-structured interviews and described how survey data were being used with the expectation that this might increase survey response rates.

### Semi-structured interviews

Convenience sampling was used to select participants. City Hosts were invited to participate in interviews via the Monitoring and Evaluation survey and further communications which came from their management team. Invitations (text and videos of the research team) were also posted on the private City Host Facebook group. Interested City Hosts approached the research team by email or telephone and were sent written participant information and consent forms. Once a signed consent form was returned to the research team, a mutually convenient time for the interview was identified and this was conducted by telephone, face-to-face or via Microsoft Teams. British Sign Language support, via the University of Warwick, was made available (as needed). Participants were allowed joint interviews (i.e. two City Hosts and the interviewer) if they preferred this format. Interviews were conducted by all four authors.

The interview guide was developed by the research team, and trialled with early participants before being used with the remaining participants (Supplementary file). The main questions asked City Hosts to describe their experiences of volunteering and the impact volunteering has had on them. The research team jointly reflected on the data as they were collected. Thematic saturation was being reached by the time 15 participants had been recruited, however the interviewees up to that point had been similar in age, and fully committed to the volunteer programme. At this point, targeted invitations were sent to City Hosts who were either aged < 35 or had less experience of City Hosting to encourage participation by under-represented groups. Additional interviews with these less well represented groups did identify further themes, but again saturation was reached before the study closed.

Interviews were audio-recorded and transcribed verbatim. We used methodology based in content analysis in which interviews were analysed deductively using the theory of change presented in Fig. 1 as a starting point, and also inductively – in order to capture additional themes of interest present in the data that were not within the previously developed theory of change. Analysis of the interview data was facilitated by NVivo (March 2020 release) and completed by IG and OO.

Some themes were amalgamated where they comprised similar concepts and quotes during the process of writing up this research.

### Survey data

The Monitoring and Evaluation team sent an online survey to all active City Hosts in August 2021, November 2021 and April 2022. The Monitoring and Evaluation surveys captured brief qualitative data via an open text box which asked City Hosts to ‘please give us your thoughts about volunteering for City of Culture and the City Host programme so far?’. The Monitoring and Evaluation team conducted this survey for the purpose of understanding how the volunteer programme was running, and to help them demonstrate that they were meeting programme goals. The research team were not involved in designing this question, or in survey administration, but were granted access to the data for secondary analyses. Answers to this question were imported into Microsoft Excel and coded using the framework developed from deductive and inductive coding of the interview data by MW.

### Participant checking

Interview participants were sent a copy of the draft study report and given an opportunity to provide feedback on the findings. Participants and other City Hosts were also invited to a study dissemination event where study findings were presented and discussed.

## Results

Overall, 27 City Hosts took part in interviews between December 2021 and May 2022. Of these, 13 identified as men and 14 as women. The youngest participant was in their twenties and the oldest in their eighties. Although participants were not directly asked, they shared information on personal characteristics during the interviews that demonstrated that the sample were diverse in terms of employment status, disability status, ethnicity, and marital status. Participants also disclosed various relationships to Coventry and the UK (in terms of migration and length of residence). All interviews were between 30 and 60 min in total.

Overall, 182 City Hosts took part in the survey in August 2021, 263 in November 2021 and 251 in April 2022. However, the number included in our analyses is lower due to some non-completion. Approximately 180 free text survey responses were analysed from each of the three City Host surveys. This means a considerable number of the 1515 fully trained City Hosts did not respond to the surveys and it is likely that those are City Hosts who had other priorities or commitments which did not allow them the time to complete the surveys. Those who responded are likely to have included those with the strongest views on the City Host programme. Survey participants were mainly women (63.9% in August 2021 − 64.7% in April 2022), mainly white British (71.9% in November 2021 − 72.7% in April 2022) with between 17.7% (August 2021) and 25.5% (April 2022) declaring a health problem or disability which limited their day-to-day activities and has lasted or is expected to last at least 12 months. Participants took part across the age groups, from 16 to 24 years to 75 + years with highest participation in 45–54, 55–64 and 65–74 year age-groups. Generally, survey participants reported similar experiences and perceptions as the interview participants, although there were some themes we did not locate within the survey data, there were no new themes arising from survey data that were not discussed by interview participants. During their interviews, City Hosts spoke of positive wellbeing impacts of volunteering, which was also reflected in survey responses. City Hosts talked about feeling greater happiness ‘Having those experiences has definitely made me feel sort of happier’ (P003), ‘I smile every time I talk about doing the hosting’ (P009); satisfaction ‘it’s quite fulfilling’ (P008); and better quality of life ‘I spend my whole life a lot better, I’m not frustrated and angry all the time’ (P007). Reduced anxiety and less depression were also mentioned by City Hosts although mainly indirectly ‘The confidence building… wouldn’t say it’s stopped me from overthinking things because I still do that, but just yeah, I guess I, I panic less’ (P032), and a survey participant recalled ‘It’s great for one’s mental well being’.

Theoretical ‘mechanisms of change’, the ways in which wellbeing might be impacted by volunteering, were supported by the qualitative data. These were (i) connecting with others, (ii) feeling appreciated, (iii) doing something purposeful and meaningful, (iv) developing and using skills and experiences, (v) role and group identity, (vi) enjoyment, (vii) structure, routine and distraction, (viii) exposure to outdoors and nature, (ix) role demands and (x) exposure to culture. Table 1 presents these mechanisms of change along with relevant quotes showing how participants brought these into discussion. The most frequent mechanism of change discussed was ‘connecting with others’. This included making connections with other City Hosts, as acquaintances, as well as deeper friendships and even romantic relationships. Several City Hosts talked about the diversity of the people they had connected with through the programme (also reflected in the quotes in Table 2, relating to better relationships). It also included connecting with the general public through the role. It was also clear from the data that City Hosts had a very strong identity, and that this identity had a key role in making them feel united, often referred to as a ‘family’. The only mechanism of change which had mixed positive and negative testimony coded to it was ‘role demands’. Volunteers liked the flexibility that the City Host programme offered, in terms of commitment and also the types of roles with many volunteers talking about their favourite roles, and the benefits of being able to choose to be a team leader or not. However, there was also disappointment when they felt they had attended events where they were not needed. Finally, a mechanism of change emerged from the data which was not included in the theory of change which was titled ‘exposure to culture’. City Hosts reported that the opportunity to take part in the city’s cultural events played a part in them accruing benefits from the programme. For some, attendance at cultural events was part of their lives before UK City of Culture 2021, but this was an opportunity to take part to a greater extent. For others, they might not have been exposed to some types of culture previously but came into contact with it via volunteering and appreciated exposure to something they wouldn’t have chosen to see (or had the opportunity to see) in other circumstances.

**Table 1:** Mechanisms of change

Theme	Quotes from the Interviews	Quotes from the surveys
Connecting with others	*To connect with people, so to build a network, because I no longer have a a network in the UK… I lived in in [Country] that 20 years and in [Country]… So it was to also make connections with people. It’s also to to meet new people that are travelling and hear their stories.* (P026) *We, we’re so good friends and not just ladies. There’s all of us you know, students, male, female and it’s amazing…, just getting to meet people again, you know. Even if it’s the hosts or the public out there, it’s just-just really good. It’s just like I said, it brings me out of my shell and you know, it’s very uplifting.* (P022)	*It’s been lovely meeting new people of all walks of life and met some great fellow hosts too.*
Feeling appreciated	*They do little rewards have like sort of 10 shifts you get a bronze award… I think there’s little things that do make you feel appreciated, and I, I and I do think any communication from them is always really positive.* (P014) *I think what what’s been great some days it’s people come up to you and say oh you know you city hosts, you’re doing a great job and that’s just the general public. Which is lovely. Or, you know, they’re really appreciative of if they’ve asked the way to something or they’ve asked you some information about about something that you’ve been able to tell them about.* (P025)	*It’s good to see the food/hot drink offer too. Little measures like this make the thought of being outside in the cold for long periods more doable, and to feel appreciated.*
Doing something purposeful and meaningful	.*and I just felt afterwards when I got home, I just thought, well, that’s just really nice. You know those people. They probably needed to have that conversation.* (P003) *The sense that I’ve helped out the people, but also the city and the City of Culture and the events and made [event] run a lot more smoothly.* (P006)	*volunteering for city of culture is life satisfaction, is good, doing good for others and the community, which provides me a natural sense of accomplishment.*
Developing and using skills and experiences	*Oh yes, yes it has had in terms of just thinking about what my employment skills are. Yeah, so that that’s made me think about other job roles that I might be more interested in future. Yeah so promoting Coventry or or or doing doing something along those lines.* (P003) *Well, I I do think as I said before, it is important to stay in the world and to and to keep learning. I don’t think you are ever too old to learn. And you can’t not learn because so much is going on. And I’ve learned more about uh computers the kind of technical side, if you like, than I knew before…But I am more kind of tecchie aware than I was which is really good.* (P012)	*I am also grateful for being given the opportunity to attend courses such as Disability Awareness.*
Role and group identity	*Just proud that you’re doing something and like belonging to like a team. People that are all together. No, you get yeah when you’re wearing the uniform like you’re proud to wear it. Like policemen… Well, no, it’s real real pride.* (P007) *The other thing that surprised me is that people, if you take the talk time to talk to them, and sometimes because you’ve got the uniform on, will tell you things they wouldn’t have told you otherwise. ‘cause, I think it has been quite successful and people have got used to seeing us around in our blue uniforms and will come and talk to us now.* (P012)	*I feel our [City Host] reputation is strong!!!* *I am honoured to be part of the volunteering programme. It’s good to be part of a team whilst doing my bit.*
Enjoyment	*We have good fun. There’s a bit of banter, most of my working life’s been in a factory so I know all about banter and um. Yeah, we’ve had some good fun and I’ve worked. I’m working with some nice people. And yeah, it’s just a pleasure to be doing it to be fair.* (P005) *enjoyed them all* (P013)	*I have enjoyed the shifts, and have attended a number of different events.*
Structure, routine, distraction	*It’s probably doing me good ‘cause I’m trying not to let my studies overtake everything so like my whole weekend becomes like I’m working, uni and then study all over the weekend. So actually it’s probably just good to put a few shifts in and just go self-care really isn’t it?* (P14) *I think volunteering is a way of being outside yourself for a while and thinking about somebody else. So if you-you need to be away from the situation you’re in, uh, being that you’re retired and on your own, or if you’re married and you just want a break from your partner, I think it’s good to do some volunteering because it takes your mind off of your situation.* (P020)	*it seemed a great way to finally get out of the house and have some purpose to my weeks which the pandemic had stopped.*
Exposure to outdoors and nature	*We’ve done a lot of walking around on the shifts, so that’s always good. Lots of lots of steps, most of them being out in the open air, so it’s always useful to get out and about.* (P008) *It’s nice to actually get out into the fresh air.* (P016)	
Role demands	*You could be a leader, or you could be a host. You could take responsibility, you didn’t have to.* (P020) *There was one there was one at the Coventry Cemetery. It’s good to walk around it and get to see it, but then you just sitting about doing nothing. There was very little footfall on it. The lack the lack of things to do at some of the events. Like I said you are just basically present, it’s not a lot of hands on, you know it’s a bit boring. You come out and spend 4 h there, you know, it’s sort of like watching paint dry.* (P002)	*At some events I feel that I do not have enough to do, or that I am an extra body. Other times I am busy and feel I am helping. The latter is preferable.*
Exposure to culture	*The richness of what I’m learning about the city, the opportunity to attend some of these events… And so you know, those are things that otherwise I probably wouldn’t attend. Uhm, especially when I wasn’t working, because if I financially, I couldn’t afford it.* (P026) *And that particular one was great because I got to talk to the artist because he was there and actually talked through his different paintings and what they meant and also what was happening with them after.* (P032) *it’s nice to be involved in it in a way that I can afford to be involved in it if that kind of makes sense.* (P014)	*I was amused by the rich history, wonderful people and hidden talents that Coventry had hidden.*

**Table 2:** Intermediate outcomes

Theme	Quotes from the interviews	Quotes from the surveys
Purpose, identity and values • Increased sense of purpose and meaning of life • New/developed sense of identity • Expression of altruism/giving back	*I want to be the person that I am now. More looking out on life… Looking positive for the future.* (P007) *I’ve seen people blossom, myself included.* (P009) *Well, I’d rather help somebody than hinder somebody, um. That might have been not been too apparent when I was younger…* (P005) *You know, we’ve got this uniform now and I would like to be a part of the city and be able to do to do more* (P016) *It feels like I’m giving back* (P013) *I’m just saying that sort of purposeful thing though. You know, I feel have contributed a little bit* (P006)	*Taking me out of my comfort zone and opening my eyes to the wider world.* *Really enjoying the opportunity to help my home City, volunteering is a great opportunity for me to give something back to my home town*
Relationships • Increased social connectedness • Increased sense of belonging/feeling part of something	*You know, we’re obviously the Coventry people. I’ve integrated with the, you know, hell of a different community, a young community, very diverse community*. (P028) *I’ve met a couple of people I worked with and lost in touch and there they were. I couldn’t believe it. So that was amazing. And one lady, I said, ‘I know you! I know you! I know you!’.* (P022) *meeting other people and sort of keeping in touch with them. So certainly there are people who I have built friendships with like [name].* (P017) *I I just think being part of something that is a unique experience within the city for the year.* (P008) *We might not all feel the same about the hosting but I think it’s bringing everyone along the journey and encouraging people to be part of it.* (P009) *Being part of a community. And not just sit not just sitting and doing nothing but being being part being part of something. You know, just sort of say well, I was part, you know. Of City of Culture I was I was part, I was part of it, which is what I wanted.* (P016) *So there’s a good sense of camaraderie… ‘cause even though we’re all doing it for unknown reasons. You know we’re all doing it for one cause which is for the City of Culture.* (P018)	*It has been a wonderful experience. I feel that I have a new family.* *I have met some great people and made a lot of new friends too*
Personal growth and development • Increased self-efficacy • Increased self-esteem • Increased confidence	*I think as a confidence builder it has been it has been good it has been, this has forced me to try out new things, so I would be less, I’ll be less fearful for trying out stuff in the future.* (P018) *I guess I’m just more open to talking to people.* (P033) *I think it’s had a really positive effect. Like I said on my wellbeing and so yeah, just going out and meeting more people… In terms of seeing people and feeling better and feeling, and I think with my confidence as well.* (P003) *I would say um so yeah, just, especially recently with doing more shifts that confidence has just increased because just through the the kind of experience and the like, almost like flooding myself with having to be in those situations.* (P032)	*I have enjoyed being a City Host. As someone who has autism for me it has helped me come more out of my shell.* *I’ve really enjoyed it but I’m getting a bit tired now.*

Intermediate outcomes suggested in the Theory of Change developed by What Works Wellbeing were also borne out by testimony from the City Hosts through both interviews and survey data [Table 2]. Not all of these applied to all the City Hosts, and many City Hosts explicitly stated that some of these did not apply to them, for example:

‘*I think I’ve always been a friendly person. I’ve always been interested in people so I can’t say that’s changed me. So I’m not really sure. I’m not really any more confident I don’t think than I was before.*’ (P029).

However, most City Hosts reported one or more of these intermediate outcomes, either (i) a contribution to their purpose, identity, and the meaning of their life, (ii) better relationships, or (iii) personal growth and development as a consequence of taking part in the volunteering programme. Table 2 provides examples of quotes that support these intermediate outcomes being part of the pathway between volunteering and wellbeing outcomes.

Again, this was overwhelmingly positive, with these intermediate outcomes leading to the overall benefits volunteers expressed in terms of their wellbeing. However, there were some very specific negative incidents identified which contributed to City Host stress. These related to difficulties booking shifts and/or feelings of unfairness relating to the booking system, some antagonism between City Hosts and paid staff in one venue − which one City Host felt was not well handled by City of Culture management − and stress relating to a specific event in which the volunteer was not protected from content that was distressing to them. It is worth noting that even these sources of stress did not undermine the overall positive impact of being a City Host for those reporting these issues, although they may have done for others who did not come forward for interviews.

City Hosts reported several barriers and facilitators to gaining the benefits of volunteering. These included individual circumstances, relating to employment, dependent family, and medical problems. However, the City Host programme offered flexibility for volunteers to choose exactly what they committed to, and this, as well as the general view that volunteering support and management was good, allowed them the chance to volunteer despite challenges. COVID-19 was mentioned by every interviewee and also by some survey participants. Volunteering was described by many as a way of re-entering society after the lockdown and restrictions, often facilitating re-engagement and re-connection with other people. Precautions (e.g. mask wearing and outdoor shifts) allowed some City Hosts to confidently volunteer despite COVID-19 concerns. Table 3 shows the key influencing factors illustrated by key quotes.

**Table 3:** Key Influencing Factors, Drivers and Barriers

Theme	Quotes from interviews	Quotes from Survey
Individual circumstances	*So I initially, I mean [I completed] the application last year before the City of Culture year was awarded, but there are some delays with the training because I had to attend face to face training, but I couldn’t make the slots. And then in the end I ended up doing the online training… there was lots of delay, in my end ‘cause I couldn’t do the face to face training.* (P023) *So ultimately I’m having to fit it round whatever work wants, so I know some people if they are retired they have a bit more time to add to hundreds of shifts, unfortunately, I’m not in that position*. (P027) *As as I started, I got a job. So my intention was to be a team leader. And because I got work, I decided in it, you know, let me let me first will get priorities in the job and see where that takes me. And so I I haven’t taken up the role of team leader. Because my work as a consultant means that I travel and I can’t, I can’t then commit two things in the week I can only commit weekends.* (P026)	
What volunteers do, how much and how often	*I think sometimes when I volunteered before for things, sometimes when people know that, I am, you’re willing to give your time. Sometimes there’s an expectation.* *They want you to give more and more time if they know that you’re free and available… That’s one of the things I like about this programme. It is very flexible. And yeah, you can fit it around work and family.* (P003) *I didn’t go for the Team Leader because I didn’t really want the responsibility. I just wanted to enjoy the experiences. Rather than getting involved with dealing with issues, you know problems and things. So I just decided to do that City Host.* (P029) *I really like that I could be anywhere I want. I could pick any shifts I want as much time as I want.* (P033) *Um, actual volunteering a lot of the time, you’re not doing a lot. Usually the volunteering like we do [in other settings] is hands on and more, um, yeah, more going on. I’m doing the vaccine clinics at the moment so we’re busy doing that but, um, with the City of Culture a lot lately is standing about. It might be 3 − 4 h, it’s not not doing a lot.* (P002)	*It has been a great opportunity although I haven’t done any hosting yet. I have some booked at the end of the week.*
Volunteering support and management	*So probably my biggest sort of like, um, minus is the is the way that the shifts are given out.* (P001) *Now, the quality of the Team Leaders in the in the Coventry City of Culture is nowhere near the quality is of the Coventry Ambassadors ‘cause they haven’t had the training and some of them it didn’t even do a shift before they become a Team Leader.* (P002) *And I cancelled a couple of sessions and the admin team were absolutely fantastic. I did tell them the reasons why. But they were brilliant and very supportive because they realise it doesn’t have to be someone being ill with COVID… So in that sense there’s no judgment it’s just thanks as long as you let them know, because that’s the most important thing.* (P009) *Within City of Culture, people said at the start of December they would investigate the whole matter and report back, two months and absolutely nothing.* (P004) *I think that they have done well that if there’s been a problem, they’ve tried to address it.* (P029)	*Excellent support from the City Host team, love that there are specific social events for the Hosts too.*
COVID-19	*2020 was a tough year from going from being in the office five days a week to going on to furlough. Everyone has struggled through the pandemic in one shape or form. It’s not. No one’s immune, no one’s not been affected in some way.* (P009) *I’ve never once felt unsafe doing it. I’ve always felt you know the right precautions has been in place. And at the, specially when you’re like outside and stuff, and in doing roaming shifts, that’s definitely sort of, you know, I don’t feel like I’m in any sort of danger of catching it and to be fair, everybody is sensible, even doing City Hosts shifts with various people, people sort of say no, no we have to do lateral flow tests, we have to wear masks.* (P018)	*It has been a great way to start getting back into the world/socialise after covid.*

## Discussion

Our analysis suggests that taking part in the City Host programme was at least partly responsible for improving wellbeing among City Hosts. This is in line with the wider literature [2, 14]. The qualitative data collected supported all the theoretical pathways to better wellbeing in a pre-existing evidence-based theory of change linking volunteering to wellbeing [Fig. 1]. The qualitative data also supported many of the themes that emerged in previous studies of volunteers engaged in mega-events, for example increased confidence, expanded friendships and bridges to diverse groups, skills and employability, volunteer legacy and pride in city [6]. City Hosts also reported an additional pathway, not included in the Theory of Change developed by What Works Wellbeing, in which exposure to culture improved their wellbeing. Other studies have noted that volunteers at cultural events have improved wellbeing and suggest that exposure to the activities (which they enjoy) plays a role in this [15] and that the exposure to culture is considered one of the motivating factors for future volunteering [16, 17]. Exposure to culture is also generally associated with wellbeing ( [18].

A key strength of this study was the inclusion of interviews with City Hosts with a variety of personal characteristics, with the interview guide allowing in-depth conversations which captured their personal perspectives and experiences of taking part in the volunteering programme. Analysis of qualitative data collected through the monitoring and evaluation survey allowed us to triangulate our findings with data from a larger sample of City Hosts (although also unlikely to be representative of City Hosts in general). These data supported the themes found in the interview data. We provided participants with the opportunity to feedback on our findings, and received positive feedback, including one written comment in response to the draft study report “As a City Host, I think the overall findings closely track my views of the programme, and anecdotal views from some city hosts I have spoken to; so I feel that my views have been represented in your research”. We are aware, though, that many City Hosts did not come forward to take part in an interview, and we did not capture many views of younger City Hosts (particularly those currently studying at the local universities) and City Hosts who were fully trained but did not engage with more than a few shifts. This may have been in part due to our recruitment strategy in which the advertisement was not seen by them, or if seen, the opportunity to speak about their City Host experience did not appeal to them.

Our findings provide some evidence of what might work well when designing volunteering programmes. Firstly, while two key mechanisms of change are discussed below, it was notable that qualitative data referred to every one of the mechanisms of change within the theory of change [Fig. 1]. By designing a volunteering programme with every mechanism of change in mind, it may be that benefits are greater and felt by more volunteers. In terms of key mechanisms of change, those that were most frequently mentioned: were:

1. Opportunities to make social connections: one of the strongest themes arising from our qualitative data related to the mechanism of change ‘connecting with others’ and intermediate outcome ‘increased social connectedness’. Volunteers reported both friendships with other City Hosts, as well as benefits derived from chances to have conversations with a more diverse circle than they usually come into contact with and members of the public. By ensuring that volunteers have the chance to meet other people through volunteering (including other volunteers, as well as the public or other beneficiaries of the volunteering activity), this maximises the opportunities to foster social connectedness that was so valued by the City Hosts.
2. A strong role and group identity: another key theme arising from the qualitative data was around the strong identity City Hosts had. This related to the mechanism of change ‘role and group identity’ and to the intermediate outcome ‘increased sense of belonging’. For the City Hosts, the uniform played a role in this, making City Hosts visible at many events across Coventry, helping to build a reputation and identity.

There are questions about whether volunteering benefits people with a range of characteristics and circumstances [19, 20]. The City Host programme allowed volunteers to match their commitments and activities to their available time and energy. This was discussed by many as facilitating their participation in the programme, as well as ensuring that what they were doing fitted in with their wider lives. This suggests that where other volunteer programmes can offer flexibility it will promote volunteering for people with a wide range of personal circumstances, while protecting volunteers from burn-out. This may reduce inequalities in benefits that has been reported as a concern in previous work on volunteering at mega-events [21].

We also noted potential barriers to wellbeing benefits of volunteering. Firstly, overbooking volunteers or recruiting volunteers without there being clear responsibilities for those turning up was something the interviewees and survey respondents were unhappy about. A lack of responsibility when volunteering is likely to diminish the intermediate outcomes of ‘sense of purpose’ and ‘expression of altruism’ that are needed to realise positive wellbeing impacts. Secondly, some volunteers discussed difficulties booking on to shifts, or the perception that some volunteers were able to get more of the highly sought-after shifts. Where necessary, considering how shifts/activities or responsibilities can be distributed fairly, could reduce the stress caused here.

## Conclusion

Our study suggests that the City Host programme increased City Host wellbeing and that this was achieved through a number of mechanisms, particularly through increasing social connectedness and increasing a sense of belonging. We have identified strengths of the City Host programme that other volunteer programmes could emulate to promote positive outcomes for their volunteers, such as flexibility around commitments and duties. We also identified barriers to wellbeing benefits, including where volunteers felt they lacked responsibilities and relating to fair access to and distribution of shifts.

### Electronic supplementary material

Below is the link to the electronic supplementary material.

Supplementary Material 1

## Data Availability

The datasets used and/or analysed during the current study are available from the corresponding author on reasonable request.
