# Correction: How and to what extent did the Coventry city of culture ‘city host’ volunteer programme affect the volunteers’ mental wellbeing? A qualitative study

**DOI:** 10.1186/s12889-024-17949-5

**Published:** 2024-02-15

**Authors:** Maxine Whelan, Iman Ghosh, Lauren Bell, Oyinlola Oyebode

**Affiliations:** 1 Institute for Health and Wellbeing, Coventry University, Coventry, UK; 2 Warwick Evidence, University of Warwick, Coventry, UK; 3 Queen Mary University of London, Wolfson Institute of Population Health, London, UK

**BMC Public Health (2023) 23:2044**

10.1186/s12889-023-16862-7

The original publication of this article contained a typo in the title. The incorrect and correct information is listed in this correction article, the original article has been updated.

Incorrect

How and to what extent did the Coventry City of Culture ‘City Host’ volunteer programme effect the volunteers’ mental wellbeing? A qualitative study.

Correct

How and to what extent did the Coventry City of Culture ‘City Host’ volunteer programme affect the volunteers’ mental wellbeing? A qualitative study.

