# Supplementary material for: How and to what extent did the Coventry City of Culture ‘City Host’ volunteer programme affect the volunteers’ mental wellbeing? A qualitative study
Source: BMC Public Health. 2023 Oct 19;23:2044. doi: 10.1186/s12889-023-16862-7 (PMC10588047; doi:10.1186/s12889-023-16862-7)
Supplement: Supplementary file 1 — Supplementary Material 1 [file 12889_2023_16862_MOESM1_ESM.docx]

**Volunteer Focus Study: What is the impact of volunteering as part of Coventry City of Culture on mental wellbeing of volunteers and on civic pride**

City Hosts Interviews – Guide

Version 2.0; dated 04/11/21

Interviewer to obtain informed consent before beginning the interview. Interviewer to introduce themselves and remind participant that the interview will be audio recorded. Participants will be encouraged to share their views on their experience of volunteering as part of Coventry City of Culture and how it has impacted their mental wellbeing, sense of pride and belonging. This will involve reflecting on their whole experience of volunteering, including any negative perceptions of the experience.

1. What is your experience of volunteering as part of Coventry City of Culture? (Prompts: specific roles/time commitment/good and bad/impact on relationships/impact on mental wellbeing/feelings of pride/feelings of belonging)

Probing questions:

- Why did you choose to be a volunteer?
- When did you start? What have you been involved in? How much time do you volunteer and how frequently?
- Did you know other people who were volunteering?
- How would you describe volunteering to others unfamiliar with it?
- How have other people reacted to you volunteering?
- Have you sacrificed anything to be part of Coventry City of Culture? (Interviewer to consider: Role demands)
- How did you find volunteering during Covid-19? (prompts: What happened?)
- What has been the most significant experience so far?
- What has surprised you most about volunteering? What are the differences between what people generally think about volunteering and the actual experience of doing it?

1. What impact has volunteering had on you? (prompts: health/relationships/communication/employment/connectedness/pride/belonging)

Probing questions:

- Consider the impact of volunteering on you, your health and your situation. What has been most significant? In what ways? Give examples.
- How did you hope volunteering might help you? What did you hope to achieve from volunteering?
- What impact do you think volunteering has on you? (prompts: wellbeing, physical health, sense of pride in Coventry, sense of belonging to Coventry)
- What changes, if any, have you made because of volunteering? (Prompt: employment, home life, personal relationships, leisure activities)
- Do you think you will return to your pre-volunteering self, or will you change due to volunteering experience?
- What is it about your volunteering experience that has caused these changes?
- How do you feel about volunteering in the future?
- Do you think volunteering would have a different impact on someone in different circumstances to you? What about people who are (e.g.: older, different gender, disability, marital status etc.)
